# Supplementary material for: The intersection of health and housing: Analysis of the research portfolios of the National Institutes of Health, Centers for Disease Control and Prevention, and U.S. Department of Housing and Urban Development
Source: PLoS One. 2024 Jan 29;19(1):e0296996. doi: 10.1371/journal.pone.0296996 (PMC10824422; doi:10.1371/journal.pone.0296996)
Supplement: S1 File — (DOCX) [file pone.0296996.s001.docx]

**S1 File. Coding Schema**

1. **Housing-Related Themes**
   - 1. *Built environment or Neighborhood Physical Attributes*
     2. *Individual Socioeconomic Disadvantage*
     3. *Neighborhood Socioeconomic Disadvantage or Disruption*
     4. *Homelessness*
     5. *Non-residential Status, Housing Instability or Insecurity*
     6. *Subsidized Housing, Public Housing, Housing Vouchers*
     7. *Neighborhood or Residential Segregation*
     8. *Housing remediations (to improve health)*
     9. *Housing environmental exposures and hazards*
     10. *Neighborhood Risk Characteristics (e.g., neighborhood violence or crime, neighborhood social environment, neighborhood disorder)*
     11. *Other*
   1. **Study design**
2. *Randomized Intervention Study (e.g., RCTs/clinical trials)*
3. *Non-randomized Intervention Study (e.g., non-RCTs, quasi-experiments, natural experiments)*
4. *Observational – Cross-sectional*
5. *Observational – Longitudinal*
6. *Methods Development (e.g., developing and testing new measures, tools, or analyses)*
7. *Cost-benefit Analysis*
8. *Other/Unclear*
   1. **Population (units of measure)**
   2. *Individuals*
   3. *Social Networks*
   4. *Families/Household*
   5. *Other/Unclear*
   6. **Setting/Geographic Scale of Research (units of measure)**
9. *Public Housing Complex/Authority/Projects*
10. *Communities/Neighborhoods*
11. *Cities/County*
12. *State(s)*
13. *National*
14. *Other/Unclear*
    1. **Geographic Setting**
       1. *Urban*
       2. *Suburban*
15. *Rural*
16. *International*
17. *Other/Unclear*
    1. **Population Life Stage/Age**
       1. *Infant and Toddlers (ages 0-4)*
       2. *Young Children to Pre-Teen (ages 5-12)*
       3. *Teenagers (ages 13-18)*
       4. *Children/Adolescents- Age Unspecified*
18. *Young Adults (ages 19-29)*
19. *Adults (ages 30-64)*
20. *Older Adults (ages 65+)*
21. *Adults- Age Unspecified*
22. *Comments on Age*
    1. **Race and Ethnicity**
23. *Black/African American*
24. *Hispanic/Latino*
25. *American Indian/Alaska Native*
26. *Native Hawaiian and other Pacific Islander*
27. *Asian American*
28. *Multi-ethnic/Cultural*
29. *White*
30. *Racial Disparities*
31. *Other/Unspecified*
    1. **Special Populations**
32. *Low-income*
33. *Veterans*
34. *Sexual and Gender Minorities*
35. *Persons with Disabilities*
36. *Homeless*
37. *Youth in/Aging Out of Foster Care*
38. *Immigrants/Refugees*
39. *Pregnant Persons*
40. *Justice (Incarcerated/Formerly Incarcerated)*
41. *Other/Unknown*
    1. **Health and Other Special Topics**
       1. *Substance Use/Abuse (including alcohol and tobacco)*
       2. *Mental Health*
       3. *Violence (domestic and neighborhood)*
       4. *Walkable Neighborhoods*
       5. *Obesity/Sedentary*
       6. *Diabetes*
       7. *Cardiovascular Disease*
       8. *Exercise/Physical Activity*
       9. *Asthma/COPD*
       10. *Policy*
42. *Cancer*
43. *HIV/AIDS*
44. *Other*
