# Supplementary material for: The intersection of health and housing: Analysis of the research portfolios of the National Institutes of Health, Centers for Disease Control and Prevention, and U.S. Department of Housing and Urban Development
Source: PLoS One. 2024 Jan 29;19(1):e0296996. doi: 10.1371/journal.pone.0296996 (PMC10824422; doi:10.1371/journal.pone.0296996)
Supplement: Attachment — Submitted filename: Response to Reviewers.docx [file pone.0296996.s005.docx]

Dong Liu, PH.D.
Academic Editor
PLOS ONE

December 1, 2023

Dear Dr. Liu:

I am submitting a revised version of “The intersection of health and housing: Analysis of the research portfolios of the National Institutes of Health, Centers for Disease Control and Prevention, and U.S. Department of Housing and Urban Development” (PONE-D-23-11897R1). My co-authors and I appreciate the reviewers’ positive and insightful comments. In response, we have made updates to the manuscript. We believe that we have been responsive to the reviewers and have made valuable revisions to the manuscript. I have uploaded two versions of the revised manuscript to the Editorial Manager system: one showing where we made changes and one “clean” version.

Below, we provide an itemized response to the reviewers’ comments.

Thank you for the opportunity to submit this revision for consideration. Please let me know if you have any questions. My co-authors and I look forward to receiving your feedback on the revision and decision on the manuscript.

Sincerely,

Liberty Walton, MPH, CHES

NIH Office of Disease Prevention

Email: [Libery.Walton@nih.gov](mailto:Libery.Walton@nih.gov)

Phone: 301-496-3979

PONE-D-23-11897R1: The intersection of health and housing: Analysis of the research portfolios of the National Institutes of Health, Centers for Disease Control and Prevention, and U.S. Department of Housing and Urban Development

**RESPONSE TO THE REVIEWS**

**Reviewer 3:**

**Overarching comments:** This is a very interesting paper. The focus on an analysis of NIH, CDC and HUD funding for housing and health research. After addressing two reviewers comments, the current introduction and literature review are comprehensive and up to date and the research purpose is clearly stated. The methods and results are explained and discussed well. I do not have any comments for the authors.

**AUTHOR’S RESPONSE:** Thank you very much for your positive feedback. We appreciate your review.

**Reviewer 4:**

**Overarching comments:** This article delves into the realm of home and health research, leveraging data from prominent research projects by NIH, HUD, and CDC. I find that the article presents a well-defined framework and holds significant implications for housing intervention policies and the enhancement of residents' well-being. In general, the author has effectively addressed the comments derived from the first round of review. However, in order to enhance the prospects of publication, I have additional several comments:

**AUTHOR’S RESPONSE:** Thank you very much for your supportive review. In response to your comments, we have included some revisions, which we believe address the comments and further strengthen the manuscript.

**Comment #1:** The literature review concerning home and health should be expanded. However, there is insufficient summary of relevant work.

**AUTHOR’S RESPONSE:** Thank you very much for the suggestion that the literature review concerning home and health should be expanded.  We have expanded the Introduction and referenced additional literature on housing and health. We note that Reviewer 3 commented that, “After addressing two reviewer’s comments, the current introduction and literature review are comprehensive and up to date…,” and much of this literature is also referenced in the Discussion. Given this paper is a descriptive review of funding, rather than a comprehensive literature review, we hope these new additions are satisfactory.

**Comment #2:** It would be beneficial to incorporate a discussion of globally pertinent policies related to home and health in the section of Introduction.

**AUTHOR’S RESPONSE**: Thank you for this suggestion. We highlight housing and health guidelines for the United States and the World Health Organization in the Introduction, and we added mention of recent housing initiatives in Canada and the UK. We also added additional information in the Discussion to reinforce the global relevance.

**Comment #3:** In the methodology section, it would be advantageous to provide an in-depth explanation of how the NIH, HUD, and CDC research projects can be harnessed to gain deeper insights into the dynamics of home and health.

**AUTHOR’S RESPONSE:** Thank you for this suggestion. We added text to the Introduction that indicates that the results of NIH, HUD, and CDC research both inform US policy and subsequent research efforts and contribute to public health recommendations. We also note in the Discussion section that the results offer critical evidence for program and policy decisions. The Methods section links to the supplemental file containing all the project abstracts which readers can read for more in-depth insights on the research supported by these three agencies.

**Comment #4:** The current reliance on basic statistical analysis may be perceived as somewhat simplistic. If feasible, the authors might consider constructing mathematical models to elucidate the underlying mechanisms, or provide a more comprehensive discussion regarding the suitability of these basic statistical analyses within the context of this article.

**AUTHOR’S RESPONSE:** Thank you for this feedback. The main objective of this manuscript is to describe the health and housing research portfolio across NIH, HUD, and CDC to identify research gaps and suggest future avenues of research. We’ve added language to the Methods to communicate that little is known about the combined agencies health and housing portfolios, and thus a descriptive analysis is a necessary step towards identifying areas for future research. While it may be perceived as somewhat simplistic, it is an approach that can commonly be found in the literature, including a 2021 PLOS ONE article ([Shams-White, et al. 2021](https://www.ncbi.nlm.nih.gov/pmc/articles/PMC8049352/)). We agree that mathematical models could be informative for future analyses, and we discuss this in the Limitations section.
